# Promising Approaches to Optimize the Biological Properties of the Antimicrobial Peptide Esculentin-1a(1–21)NH_2_: Amino Acids Substitution and Conjugation to Nanoparticles

**DOI:** 10.3389/fchem.2017.00026

**Published:** 2017-04-25

**Authors:** Bruno Casciaro, Floriana Cappiello, Mauro Cacciafesta, Maria Luisa Mangoni

**Affiliations:** ^1^Laboratory Affiliated to Istituto Pasteur Italia-Fondazione Cenci Bolognetti, Department of Biochemical Sciences, Sapienza University of RomeRome, Italy; ^2^Department of Cardiovascular, Respiratory, Nephrological, Anesthesiological and Geriatric Sciences, Sapienza University of RomeRome, Italy

**Keywords:** antimicrobial peptide, frog-skin, antibiotic-resistance, D-amino acids, gold nanoparticles, *Pseudomonas aeruginosa*

## Abstract

Antimicrobial peptides (AMPs) represent an interesting class of molecules with expanding biological properties which make them a viable alternative for the development of future antibiotic drugs. However, for this purpose, some limitations must be overcome: (i) the poor biostability due to enzymatic degradation; (ii) the cytotoxicity at concentrations slightly higher than the therapeutic dosages; and (iii) the inefficient delivery to the target site at effective concentrations. Recently, a derivative of the frog skin AMP esculentin-1a, named esculentin-1a(1–21)NH_2_, [Esc(1–21): GIFSKLAGKKIKNLLISGLKG-NH_2_] has been found to have a potent activity against the Gram-negative bacterium *Pseudomonas aeruginosa*; a slightly weaker activity against Gram-positive bacteria and interesting immunomodulatory properties. With the aim to optimize the antimicrobial features of Esc(1–21) and to circumvent the limitations described above, two different approaches were followed: (i) substitutions by non-coded amino acids, i.e., α-aminoisobutyric acid or d-amino acids; and (ii) peptide conjugation to gold nanoparticles. In this mini-review, we summarized the structural and functional properties of the resulting Esc(1–21)-derived compounds. Overall, our data may assist researchers in the rational design and optimization of AMPs for the development of future drugs to fight the worldwide problem of antibiotic resistance.

## Antimicrobial peptides: general features

Gene-encoded antimicrobial peptides (AMPs) are evolutionally conserved molecules produced by almost all living organisms (e.g., bacteria, fungi, higher eukaryotes including humans, Ageitos et al., 2016). As part of key effectors of the innate immunity, they act as a sudden response against a multitude of microorganisms before the adaptive immune system comes into action (Boman, 1995; Hemshekhar et al., 2016). Despite their different length and secondary structure ranging from an α-helix, a β-strand, a loop, or an extended conformation in hydrophobic environments, most of them share an amphipathic and cationic character at neutral pH (Powers and Hancock, 2003). These two properties are crucial factors, especially for the mechanism of action of α-helical AMPs, which is generally based on the perturbation of the target microbial membrane (Bechinger and Gorr, 2017). More specifically, it consists in an initial electrostatic interaction between the positively-charged AMP and the negatively-charged components of the microbial cell surface, such as lipoteichoic acids in Gram-positive bacteria, or lipopolysaccharides (LPS) in Gram-negatives, to finally reach the plasma-membrane. This is then destabilized by pores formation/local cracks or disintegration in a detergent-like manner, with consequent cell death (Hall and Aguilar, 2010). Peptide-membrane interaction is the most important step controlling the selectivity of AMPs toward microbial membranes, which are much richer in anionic phospholipids compared to those of mammalian cells mainly made of electrically-neutral (zwitterionic) lipids (Oren et al., 1999). However, peptide-membrane interaction is dictated not only by the peptide's cationicity, but also by other physiochemical parameters of AMPs encompassing their length, hydrophobicity, amphipathicity, and helicity (Marín-Medina et al., 2016). Remarkably, unlike conventional antibiotics, this non-specific mechanism of action of AMPs very rarely induces resistance (Bechinger and Gorr, 2017) and makes them an interesting class of molecules for the development of new antimicrobial compounds (Mazer-Amirshahi et al., 2016). To date, thousands of AMPs have been characterized from a variety of natural sources as well as their synthetic derivatives (Liu et al., 2016). Noteworthy, an increasing number of AMPs has already entered into advanced stages of clinical trials for topical treatment of different types of infections. Nevertheless, several limitations can hinder their development as new therapeutics (da Cunha et al., 2017). Among them: (i) the cytotoxicity at concentrations slightly higher than antimicrobial dosing; (ii) the low peptide biostability due to fast proteolytic degradation; and (iii) the inefficient delivery to the target site at effective concentrations (Fjell et al., 2012). Nowadays, thanks to the progress in computational studies and nanotechnologies, it is possible to circumvent these issues. In this mini-review article, after a brief overview on amphibian AMPs and the structural/functional relationships of the frog skin-derived AMP esculentin-1a(1–21)NH_2_, Esc(1–21), we will mainly focus on the principal approaches that have been used to optimize the biological properties of Esc(1–21): (i) substitution by non-coded amino acids and (ii) conjugation to inorganic nanoparticles.

## Amphibian skin antimicrobial peptides

Among natural storehouses of AMPs, frog skin is one of the richest (Conlon, 2011b). The expression of genes encoding for these peptides in dermal serous glands is induced upon contact with microorganisms (Mangoni et al., 2001); and the produced AMPs are stored within granules that are released onto the skin surface in a holocrine mechanism after stress or tissue injury (König et al., 2015). It was first discovered that amphibian AMPs do not only protect the host from invading microbial pathogens, but also regulate the animal's natural flora (Simmaco et al., 1998); a trait which has then been confirmed also for human AMPs (Mangoni et al., 2016). Over the years, since the discovery of magainins from the skin of *Xenopus laevis* (Zasloff, 1987), an increasing number of AMPs has been identified from different Anuran species (Coccia et al., 2011; Conlon, 2011a). In particular, from various *Rana* genera, a large number of AMPs has been isolated and characterized. On the basis of their common structural features, they have been classified into several families encompassing brevinins-1, brevinins-2, nigrocins, temporins, esculentins-1, and esculentins-2 (Conlon et al., 2004).

### Esculentin-1a(1–21)NH_2_: synthesis and characterization

All members of the esculentin-1 family have a primary structure composed of 46 amino acids and contain a C-terminal hepta-membered ring stabilized by a disulfide bridge (Mangoni et al., 2015). They adopt an amphipathic α-helix structure in membrane mimicking environments and have a net charge of +5 at neutral pH (Wang et al., 2016). AMPs belonging to the esculentin-1 family were initially isolated and purified by reverse-phase high performance liquid chromatography (RP-HPLC) from the cutaneous secretions of *Pelophylax lessonae/ridibundus* (previously classified as *Rana esculenta*) specimen (Simmaco et al., 1993). They have a highly conserved C-terminal half and differ by only one or two residues in the N-terminal region (Simmaco et al., 1994). Interestingly, a fragment corresponding to the 19–46 portion of esculentin-1 peptides was isolated from one of the HPLC fractions but it was devoid of antimicrobial activity, probably due to its low net positive charge (+1 vs. +5 of the full-length AMP, Simmaco et al., 1994). It was then investigated whether the antimicrobial activity was retained in the N-terminal half of the molecule. For this reason, a peptide corresponding to the 1–18 fragment of esculentin-1b was chemically synthesized and amidated at its C-terminus (Mangoni et al., 2003). Note that the C-terminal amidation is a common post-translational modification in linear AMPs from frog skin (Nicolas and El Amri, 2009). This peptide, named Esc(1–18), had a comparable antimicrobial activity to that of the full-length parent esculentin-1, but a lower hemolytic capacity (Mangoni et al., 2003).

**Figure 1:**
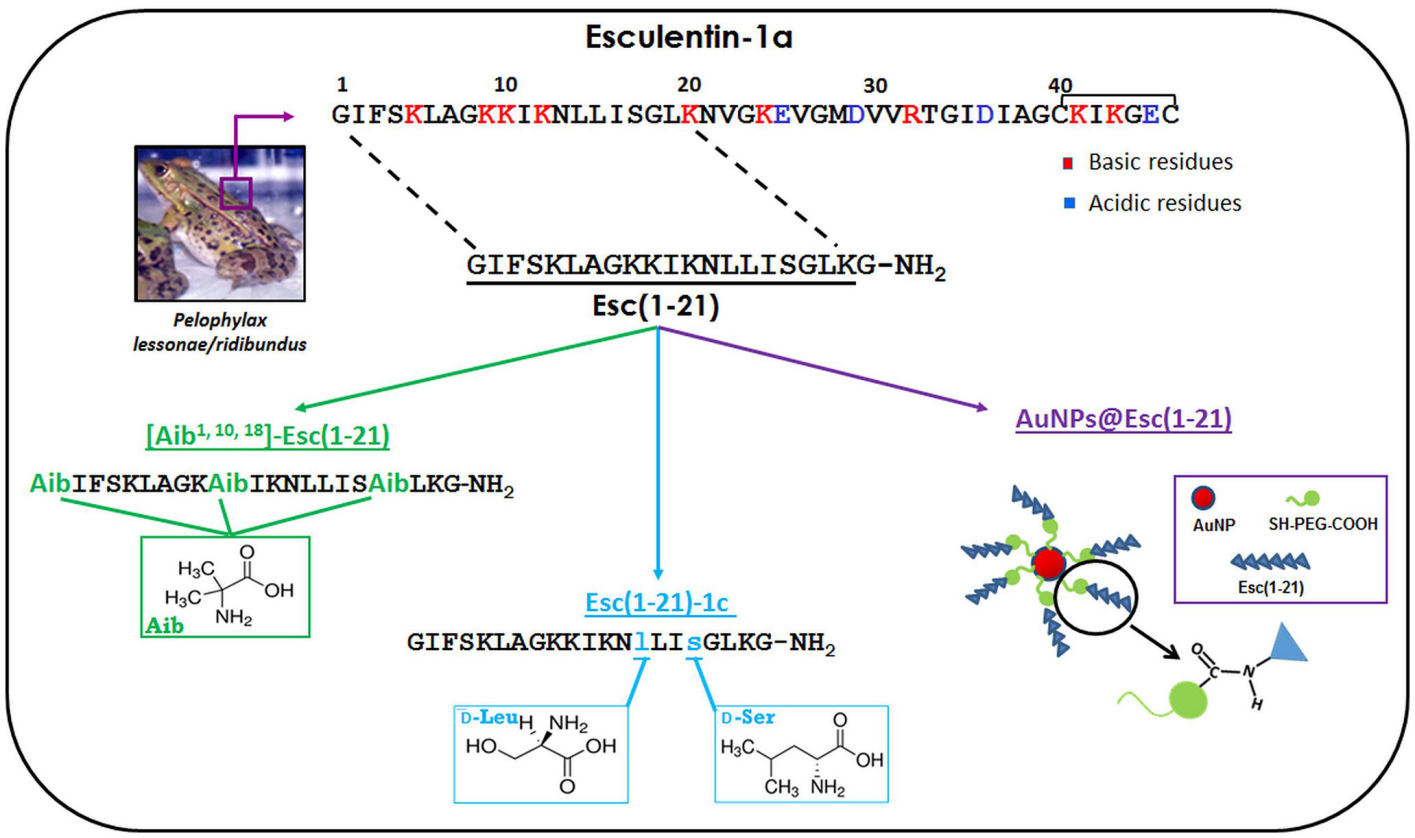
**Schematic representation of the amino acids substitutions used to design the Esc(1–21)-analogs and a representative image of the peptide-conjugated AuNPs**.

Structurally, Esc(1–18) was found to adopt an α-helix structure in lipid vesicles mimicking the anionic character of microbial membranes (Mangoni et al., 2003). It rapidly killed bacteria (e.g., *Escherichia coli*) within 15–20 min with concomitant leakage of cytosolic material, presumably due to the formation of transient membrane-breakages (Marcellini et al., 2009). Since the minimum length for a peptide in α-helix conformation to span a phospholipid bilayer (~30 Å thick) is about 20 amino acids, a longer analog named esculentin-1a(1–21)NH_2_, Esc(1–21), was further synthesized and characterized for its biological properties. Esc(1–21) shares the first 20 residues with esculentin-1a (Figure 1) followed by an amidated glycine (Islas-Rodrìguez et al., 2009). It differs from Esc(1–18) for having (i) an Ile residue at position 11 instead of a Leu and (ii) three additional C-terminal residues (Leu-Lys-Gly) which confer it a higher net positive charge at neutral pH (+6). This should strengthen the electrostatic interaction of the peptide with the negatively-charged membrane of microbial cells. Indeed, Esc(1–21) exhibited a higher antimicrobial potency than Esc(1–18) (Mangoni et al., 2015), especially against Gram-negative bacteria, e.g., the opportunistic pathogen *Pseudomonas aeruginosa* (Gellatly and Hancock, 2013). Esc(1–21) displayed a quick bactericidal activity within 15 min against both reference and clinical isolates of *P. aeruginosa* with concentrations causing 99.9% killing between 0.5 and 1 μM in physiological solution (Luca et al., 2013). Differently, a weaker activity was detected against Gram-positive bacteria, as pointed out by the higher values of minimum inhibitory concentration (MIC) compared to those recorded toward Gram-negatives (Kolar et al., 2015).

Among other interesting biological features, Esc(1–21) was found to have the ability (i) to hinder the secretion of the pro-inflammatory cytokine TNF-α from *P. aeruginosa* LPS-stimulated immune cells and (ii) to induce re-epithelialization of a pseudo-“wound” area generated in a monolayer of keratinocytes, the most abundant cells in epidermis (Haslam et al., 2014), at a faster rate than the mammalian AMP LL-37 (Di Grazia et al., 2015b). This is a relevant matter, which is not shown by any traditional antibiotic. Note that the healing of an injured infected tissue does not only require elimination of microbial pathogens but also the recovery of the tissue integrity along with its barrier function to prevent pathogens penetration.

Nevertheless, Esc(1–21) is not free from the restrictions mentioned above. In the following sections, we summarize the outcome of two different rational approaches employed to increase the biostability of this peptide and to decrease its cytotoxicity without compromising its antimicrobial efficacy: modification of Esc(1–21) by non-coded amino acids i.e., α-aminoisobutyric acid (Aib) or d-amino acids, and (ii) its conjugation to gold nanoparticles (AuNPs). All these modifications are represented in Figure 1.

#### The analog [Aib^1,10,18^]-Esc(1–21)

The Aib residue is a non-natural amino acid mainly used to increase the stability of α-helix conformation (Bellanda et al., 2001). Due to their strong helicogenicity (De Zotti et al., 2012a), when Aib residues are inserted into the primary structure of peptides, they are expected to increase their helical content and to protect them from proteolytic attack (Rink et al., 2010). The usage of Aib residues is also expected to enlarge the spectrum of activity of Esc(1–21). This is because a stabilized α-helix structure is reported to be correlated to the AMPs' activity against Gram-positive bacteria (Giangaspero et al., 2001) toward which Esc(1–21) is not particularly active. The Aib-analog was synthesized by replacing three amino acids in positions 1, 10, and 18 with Aib residues (Figure 1). This replacement was rationally designed on the basis of the following considerations: (i) an Aib residue in position 1 should prevent enzymatic degradation by aminopeptidases, while the protection of the C-terminus from carboxypeptidases would not be necessary, due to the presence of an amidated glycine in Esc(1–21) (Rink et al., 2010); (ii) Aib residues in positions 10 and 18 should contribute to stabilize the α-helix structure, due to their stronger α-helix-promoting activity when placed internally to the primary structure of a peptide; (iii) according to an ideal α-helix folding of Esc(1–21), it was possible to identify an hydrophobic face and an hydrophilic one (Biondi et al., 2016). It is known that in naturally-occurring Aib-rich peptides, such as peptaibiotics, Aib residues are located either within the hydrophobic face or at its boundary with the hydrophilic one (Toniolo et al., 1994; De Zotti et al., 2012b).

The secondary structure of both Esc(1–21) and its [Aib^1,10,18^]-Esc(1–21) was initially investigated by circular dichroism (CD) (Biondi et al., 2016) in water and two different membrane-mimicking environments e.g., sodium dodecyl sulfate (SDS) aqueous solution and trifluoroethanol (TFE). The results confirmed that both peptides adopted an unordered conformation in water and an α-helix structure in both SDS and TFE. However, at increasing concentration of TFE (from 20 to 50%) the helical content in [Aib^1,10,18^]-Esc(1–21) sharply increased with respect to the parent peptide. The helical and less flexible structure of [Aib^1,10,18^]-Esc(1–21) compared to Esc(1–21) was also confirmed by 2D-NMR analysis in TFE solution (Biondi et al., 2016).

Overall, the greater stability and content of α-helix in the Aib-analog were found to influence the biological properties of the peptide. More precisely, the Aib-analog gained an overall higher activity against Gram-positive bacteria, especially those belonging to *Staphylococcus* genus [MIC of 2–4 vs. 16–64 μM of Esc(1–21)] without losing its efficacy against Gram-negative bacteria and *Candida* species (Biondi et al., 2016). It is possible that differences in the composition of the membrane or cell wall among these microorganisms account for the different activity of the two esc-peptides against them.

However, a higher α-helicity in [Aib^1,10,18^]-Esc(1–21) resulted in increased cytotoxicity against mammalian cell lines (e.g., alveolar epithelial cells and keratinocytes). The experimental data showed that, at the antimicrobial concentrations, Esc(1–21) and [Aib^1,10,18^]-Esc(1–21) were harmless to human cells, while at higher concentrations the cytotoxic effect of the Aib-analog became clearly evident in comparison with the parent Esc(1–21) (Biondi et al., 2016). This is consistent with the notion that both α-helix conformation and its stability are crucial parameters for mammalian membrane perturbation and cell lysis, likely assisting the peptide's penetration into the hydrophobic core of phospholipids bilayers (Shai and Oren, 1996).

#### The analog Esc(1–21)-1c

With the aim to reduce the cytotoxicity and to protect Esc(1–21) from proteolytic degradation, another analog carrying two d-amino acids was synthesized: Esc(1–21)-1c. It was obtained by replacing two l-amino acids i.e., Leu^14^ and Ser^17^ with the corresponding d-amino acid enantiomers (Figure 1). This diastereomer was rationally designed on the basis of the following considerations: (i) d-amino acids are known to be “α-helix breakers” (Grieco et al., 2013) and a reduction in the α-helix content of the peptide should reduce its propensity to perturb mammalian membranes leading to cell death (Strahilevitz et al., 1994); (ii) previous studies on the shorter analog Esc(1–18) pointed out that in electrically-neutral lipid vesicles the peptide adopted an α-helix conformation at its C-terminal half. With the purpose to disrupt at least the first turn of the α-helix expected to be present in the C-terminal half of Esc(1–21) in mammalian cell membranes, analogously to what found for Esc(1–18), replacement of two l-amino acids with the corresponding d-enantiomers was carried out at position 14 and 17. Note that it was improbable that the C-terminal tail Gly18-Gly21 of Esc(1–21) folded in a stable helical conformation.

The stability of both isomers was initially examined in the presence of 10 and 30% fresh human serum after 24 h incubation at 37°C. The data revealed that in comparison with Esc(1–21) <50% of the diastereomer was degraded (Di Grazia et al., 2015a). Besides, the presence of these two d-amino acids made the peptide significantly more resistant to the proteolytic cleavage caused by both human and bacterial elastases (Cappiello et al., 2016).

When the structure of the two peptides was analyzed by CD in lysophosphatidylcholine (LPC), which simulates the zwitterionic nature of mammalian cell membranes, a loss of α-helix structure was clearly detected for Esc(1–21)-1c (Di Grazia et al., 2015a). In contrast with data obtained for the Aib-analog, this diastereomer was significantly less toxic than Esc(1–21) against mammalian cells, either circulating cells (e.g., erythrocytes, macrophages) or epithelial cells. More precisely, its LD_50_ was higher than 256 μM in comparison with a LD_50_ ranging from 64 to 150 μM for the all-l peptide toward macrophages and epithelial cells, respectively (Di Grazia et al., 2015a; Cappiello et al., 2016). Interestingly, the introduction of these two residues in the d-configuration also conferred the peptide: (i) a higher tendency than the all-l counterpart to kill *P. aeruginosa* biofilms at concentrations lower than 25 μM (despite the diastereomer had a slightly reduced bactericidal activity against the free-living form of this pathogen); and (ii) a higher “wound” healing activity *in vitro* (Di Grazia et al., 2015a).

#### AuNPs@Esc(1–21)

Amino acids replacement is not the only strategy to increase the stability of a peptide to proteolytic degradation. Moreover, this approach does not allow a peptide to overcome biological barriers (e.g., mucus, skin layers) before reaching the site of infection at high active concentrations (d'Angelo et al., 2015). A different biochemical approach to also assist drug delivery at effective concentrations is given by its conjugation to nanoparticles (NPs). This would enable not only to protect the drug from the external environment but also to increase its local concentration.

Among the various NPs produced in recent years, AuNPs have attracted most attention due to their small size, high solubility, stability, biocompatibility, and chemical inertness (Connor et al., 2005). They can diffuse through all layers of human skin (Williams et al., 2006) and because of their large surface area, they can be functionalized with a high number of molecules (Yih and Al-Fandi, 2006; Pietro et al., 2016; Soica et al., 2016). Nevertheless, only a limited number of studies has been reported to date on the effects of conjugation of AMPs to AuNPs (Rai et al., 2016). By using Esc(1–21) as a model peptide, it was demonstrated for the first time how a chemical conjugation of an AMP via polyethylene glycol (PEG) linker to AuNPs increases its antimicrobial activity while retaining its mode of action without becoming toxic to human keratinocytes. AuNPs were synthetized by the citrate reduction of gold and stabilized with a bifunctional PEG bearing a thiol and a carboxylic group. The PEG was attached to the AuNPs via a gold-thiol bond (AuNPs@PEG), while the carboxylic group was used for further derivatization with the peptide via carbodiimide-mediated coupling (Casciaro et al., 2017).

Remarkably, the obtained AuNPs@Esc(1–21) resulted to be more active than the free peptide against both planktonic and sessile forms of *P. aeruginosa*. This was indicated by the corresponding minimal concentrations causing 50% killing of both bacterial phenotypes which were found to be ~15-fold lower than those of the free Esc(1–21). This is presumably due to the higher concentration of peptide molecules at the site of bacterium-NP contact, as visualized by electron microscopy images which evidenced how these AuNPs@Esc(1–21) form clusters at various points on the bacterial surface with disruption of the membrane and leakage of cytosolic material. Otherwise, our unconjugated bare-AuNPs did not show any anti-pseudomonal activity and were not detected around bacterial cells (Casciaro et al., 2017). This is in line with the findings that non-functionalized AuNPs are harmless also to other bacterial pathogens (Williams et al., 2006) and suggests that the cationic AMP represents the driving force allowing AuNPs@Esc(1–21) to reach the target site at high concentration. In addition, AuNPs@Esc(1–21) were resistant to proteolytic degradation preserving their antibacterial activity 2 h after treatment with trypsin (Casciaro et al., 2017). Finally, AuNPs@Esc(1–21) were harmless to keratinocytes and retained the peptide's capability to stimulate migration of keratinocytes in a pseudo-“wound” healing assay. Altogether these findings make AuNPs@Esc(1–21) an attractive nano-formulation for topical treatment of skin infections (Casciaro et al., 2017).

## Conclusions

Antibiotic-resistant microbial infections cause thousands of deaths per year worldwide and this necessitates the discovery of new compounds to counter them. In this scenario, AMPs represent promising anti-infective molecules with expanding properties. However, their low biostability, cytotoxic effect at concentrations higher than therapeutic dosages and the difficulty in reaching target sites at active concentration, remain disadvantages that must be overcome. In this mini-review, by using Esc(1–21) as a reference, we have summarized how (i) substitution of natural amino acids by non-coded residues as well as (ii) peptide conjugation to AuNPs represent encouraging methodologies to optimize the biological properties of an AMP. Each synthetized analog/compound showed its own peculiarities according to its structural features (Table 1). Overall, the two different approaches should serve as an example to assist and to ameliorate the development of new peptide-based formulation for an efficient treatment of different types of infectious diseases.

**Table 1:** **Structural properties and biological features of the designed Esc(1–21)-derived compounds**.

**Compound**	Structural Properties^*^	Biological Features^*^
[Aib^1,10,18^]-Esc(1–21)	Higher α-helical content in the secondary structure	Same activity against Gram-negative bacteria and yeasts Higher activity against Gram-positive bacteria Higher cytotoxicity against mammalian cells
Esc(1–21)-1c	Lower α-helical content in the secondary structure	Higher resistance to proteolytic degradation Slightly lower activity against the planktonic form of *P. aeruginosa* Higher activity against the sessile form of *P. aeruginosa* Lower cytotoxicity against mammalian cells Greater efficacy in promoting migration of human lung epithelial cells
AuNPs@Esc(1–21)	Conjugation to AuNPs *via* PEG linker (~16 peptide molecules per AuNP@PEG)	Higher activity against both planktonic and sessile forms of *P. aeruginosa* Higher resistance to trypsin degradation Invariant membrane-perturbing activity Negligible cytotoxicity on human keratinocytes Similar ≪wound≫ healing effect

* *with respect to Esc(1–21)*.

## Author contributions

BC wrote the review article; FC prepared and assembled the figure/table; MC and MM critically revised the manuscript.

## Funding

The work was supported by grants from Sapienza University of Rome (Ricerca Università 2016). Part of the work was also supported by FILAS Grant Prot. FILAS RU-2014-1020.

### Conflict of interest statement

The authors declare that the research was conducted in the absence of any commercial or financial relationships that could be construed as a potential conflict of interest.
